# Infective Endocarditis Risk with Melody versus Sapien Valves Following Transcatheter Pulmonary Valve Implantation: A Systematic Review and Meta-Analysis of Prospective Cohort Studies

**DOI:** 10.3390/jcm12154886

**Published:** 2023-07-25

**Authors:** Akshay Machanahalli Balakrishna, Danielle B. Dilsaver, Ahmed Aboeata, Ramesh M. Gowda, Andrew M. Goldsweig, Saraschandra Vallabhajosyula, Jason H. Anderson, Trevor Simard, Aravdeep Jhand

**Affiliations:** 1 Department of Internal Medicine, Creighton University School of Medicine, Omaha, NE 68124, USA; 2 Department of Medicine, Division of Clinical Research and Public Health, Creighton University School of Medicine, Omaha, NE 68124, USA; 3 Division of Cardiovascular Medicine, Department of Medicine, Creighton University School of Medicine, Omaha, NE 68124, USA; 4 Department of Interventional Cardiology, Icahn School of Medicine at Mount Sinai Morningside and Beth Israel, New York, NY 10029, USA; 5 Department of Cardiovascular Medicine, Baystate Medical Center, Springfield, MA 01199, USA; 6 Division of Cardiovascular Medicine, University of Nebraska Medical Center, Omaha, NE 68105, USA; 7 Section of Cardiovascular Medicine, Department of Medicine, Wake Forest University School of Medicine, Winston-Salem, NC 27101, USA; 8 Department of Cardiovascular Medicine, Mayo Clinic, Rochester, MN 55905, USA

**Keywords:** transcatheter pulmonary valve implantation, TPVI, PPVI, infective endocarditis, Sapien valve, Melody valve, meta-analysis

## Abstract

Background: Transcatheter pulmonary valve implantation (TPVI) is an effective non-surgical treatment method for patients with right ventricle outflow tract dysfunction. The Medtronic Melody and the Edwards Sapien are the two valves approved for use in TPVI. Since TPVI patients are typically younger, even a modest annual incidence of infective endocarditis (IE) is significant. Several previous studies have shown a growing risk of IE after TPVI. There is uncertainty regarding the overall incidence of IE and differences in the risk of IE between the valves. Methods: A systematic search was conducted in the MEDLINE, EMBASE, PubMed, and Cochrane databases from inception to 1 January 2023 using the search terms ‘pulmonary valve implantation’, ‘TPVI’, or ‘PPVI’. The primary outcome was the pooled incidence of IE following TPVI in Melody and Sapien valves and the difference in incidence between Sapien and Melody valves. Fixed effect and random effect models were used depending on the valve. Meta-regression with random effects was conducted to test the difference in the incidence of IE between the two valves. Results: A total of 22 studies (including 10 Melody valve studies, 8 Sapien valve studies, and 4 studies that included both valves (572 patients that used the Sapien valve and 1395 patients that used the Melody valve)) were used for the final analysis. Zero IE incidence following TPVI was reported by eight studies (66.7%) that utilized Sapien valves compared to two studies (14.3%) that utilized Melody valves. The pooled incidence of IE following TPVI with Sapien valves was 2.1% (95% CI: 0.9% to 5.13%) compared to 8.5% (95% CI: 4.8% to 15.2%) following TPVI with Melody valves. Results of meta-regression indicated that the Sapien valve had a 79.6% (95% CI: 24.2% to 94.4%, *p* = 0.019; R^2^ = 34.4) lower risk of IE incidence compared to the Melody valve. Conclusions: The risk of IE following TPVI differs significantly. A prudent valve choice in favor of Sapien valves to lower the risk of post-TPVI endocarditis may be beneficial.

## 1. Introduction

**Figure 1:**
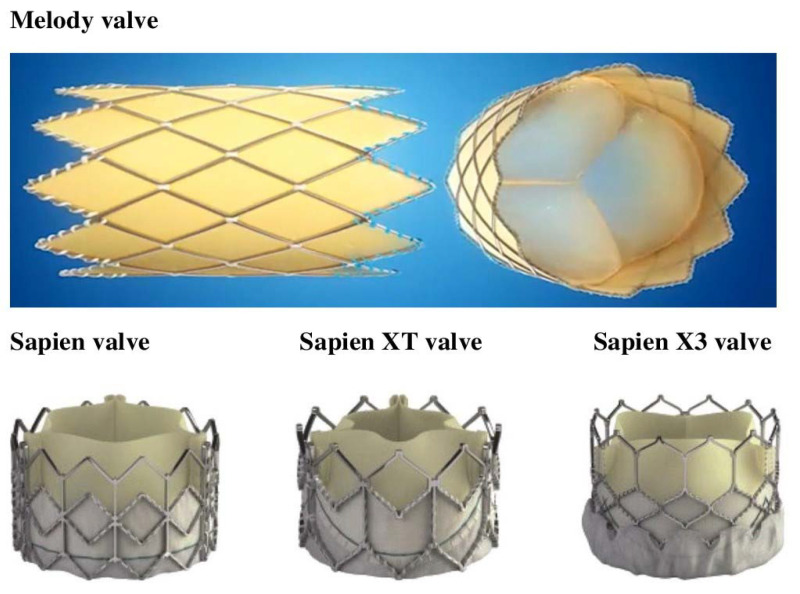
Illustration of different Melody and Sapien valves. Obtained from https://www.medtronic.com/us-en/healthcare-professionals/products/cardiovascular/transcatheter-pulmonary-valve/melody-ensemble-system/therapy-procedure.html (accessed on 19 July 2023) and https://www.edwards.com/healthcare-professionals/products-services/transcatheter-heart/transcatheter-sapien-3-valve-pulmonic (accessed on 19 July 2023).

Transcatheter pulmonary valve implantation (TPVI) or percutaneous pulmonary valve implantation (PPVI), originally reported in the late 1990s, is a major advancement in the percutaneous management of congenital heart disease (CHD) [1]. Due to the poor long-term performance of the biological valve systems, most individuals with right ventricular outflow tract disease require numerous treatments or operations throughout their lives, which makes TPVI an attractive and less invasive substitute for reoperation of right ventricular outflow tract (RVOT) dysfunction in patients with CHD [2]. Initially, TPVI was only available to patients with a defective right ventricle-to-pulmonary artery conduit; however, it has since been made available to individuals with bioprostheses, small expandable conduits, and native RVOTs [3,4]. The Edwards Sapien (Edwards Lifesciences, Irvine, CA, USA) and the Melody Medtronic (Medtronic Inc., Minneapolis, MN, USA) valves are the most commonly used transcutaneous pulmonary valve systems among the commercially available and approved valves for interventional TPVI in the United States (Figure 1) [5,6].

Although the optimal indications and timing of TPVI are unclear at this time [7,8,9], it is commonly used in patients with CHD, such as in tetralogy of Fallot, pulmonary atresia, truncus arteriosus, and following Ross or Rastelli surgery. Because of the underlying cardiac defects, prior surgeries, and the implanted valves, these patients are at a higher risk of infective endocarditis (IE) and may require prophylactic antibiotics [10]. A history of IE, immunodeficiency, a smaller conduit, and a residual valve gradient are the major established risk factors for IE [11,12]. It is uncertain if the risk of IE varies among different types of valves [11,13,14,15,16,17,18]. The study’s objectives were to ascertain the incidence of IE following TPVI and identify disparities, if any, between the two commonly used pulmonary valves.

## 2. Materials and Methods

### 2.1. Data Sources and Search Strategies

A systematic review of the published literature was conducted following the PRISMA (Preferred Reporting Items for Systematic Reviews and Meta-Analyses) guidelines [19]. A comprehensive search of the MEDLINE, EMBASE, PubMed, and Cochrane databases was conducted from inception to 1 January 2023 using relevant Medical Subject Headings and variations. The following search terms and keywords were used: “pulmonary valve implantation”, “TPVI”, or “PPVI”. An experienced librarian designed and carried out the search strategy with assistance from two authors (A.M.B. and A.A.). A complete export of the results was made to EndNote (Clarivate), where any evident duplicates were removed. Two reviewers independently screened each of the identified abstracts (A.M.B. and A.A.). Articles for qualitative and quantitative synthesis were included based on the consensus of the two reviewers. References from the included studies were examined to identify any other potential publications of interest. The senior author (A.J.) acted as the referee in cases of dispute. Because publicly accessible data were used, institutional review board approval was not sought.

### 2.2. Study Selection, Data Extraction, and Quality Assessment

Studies were included if they estimated the incidence of IE with TPVI when Melody and/or Sapien (Sapien, Sapien XT, or Sapien 3) valves were used. Prospective observational cohort, case–control, and randomized studies were considered for inclusion. Retrospective studies, systemic or narrative reviews, case reports, pediatric or animal studies, and those without data related to the outcomes of interest were excluded. Studies were also excluded if they reported using the valves of interest in the aortic, mitral, and tricuspid positions. To judge the methodologic quality of the retained records, the Newcastle–Ottawa Scale (NOS) quality score was applied to assess the quality of the non-randomized studies in the meta-analysis. (Supplemental Table S1).

### 2.3. Study Outcomes

The primary outcome was the pooled incidence of IE diagnosis following TPVI with both valves. Separate pooled incidences were provided by valve along with the difference in incidence between Sapien and Melody valves.

### 2.4. Statistical Analysis

The natural log link of study-specific IE incidences was used to obtain the pooled incidence of IE. Study-specific standard errors were based on the number of IE diagnoses following TPVI and study-specific sample sizes. Standard error was calculated as the square root of (1/*k* − 1/*N*), where *k* represents IE diagnoses and *N* represents sample size. To allow for calculation of log incidence and its standard error in studies reporting no incidence of IE diagnoses, a continuity correction was used. Although continuity corrections address concerns about excluding zero-event studies, their addition of pseudo-observations may bias conclusions in studies with small sample sizes [20]. Therefore, to minimize bias, we used a continuity correction of 0.001. Further, to evaluate any bias resulting from our use of this continuity correction, we conducted several sensitivity analyses that included a continuity correction of 0.5, the exclusion of zero-event studies, as well as estimation of random-effect Poisson and binomial regression models that allow for zero-event studies.

For primary analysis, heterogeneity among studies was quantified by I^2^; low, moderate, and high heterogeneity were defined at thresholds of 25%, 50%, and 75%, respectively. A fixed-effect model with inverse variance estimation was used for low heterogeneity. For moderate or high heterogeneity, a random-effects model with restricted maximum likelihood estimation was used. Results are reported using forest plots with inverse-linked log incidence to allow for reporting on the incidence scale. Small-study bias is shown via funnel plots. Meta-regression was conducted to evaluate the difference in incidence between Sapien and Melody valves. To account for differences in the length of follow-up between Sapien and Melody valves, meta-regression was performed using the length of follow-up as a covariate. The proportion of between-study heterogeneity explained by Sapien vs. Melody valves was quantified using *R*^2^. For the sensitivity analyses, between-study heterogeneity was quantified via I^2^ as well as random intercept variance. Model comparison was used to evaluate statistically significant heterogeneity using the likelihood ratio test. Statistical significance was indicated by *p* < 0.05.

Primary meta-analysis was conducted with the meta package in Stata v. 17.0. Secondary sensitivity analysis was conducted using SAS v. 9.4.

## 3. Results

### 3.1. Search Results

**Figure 2:**
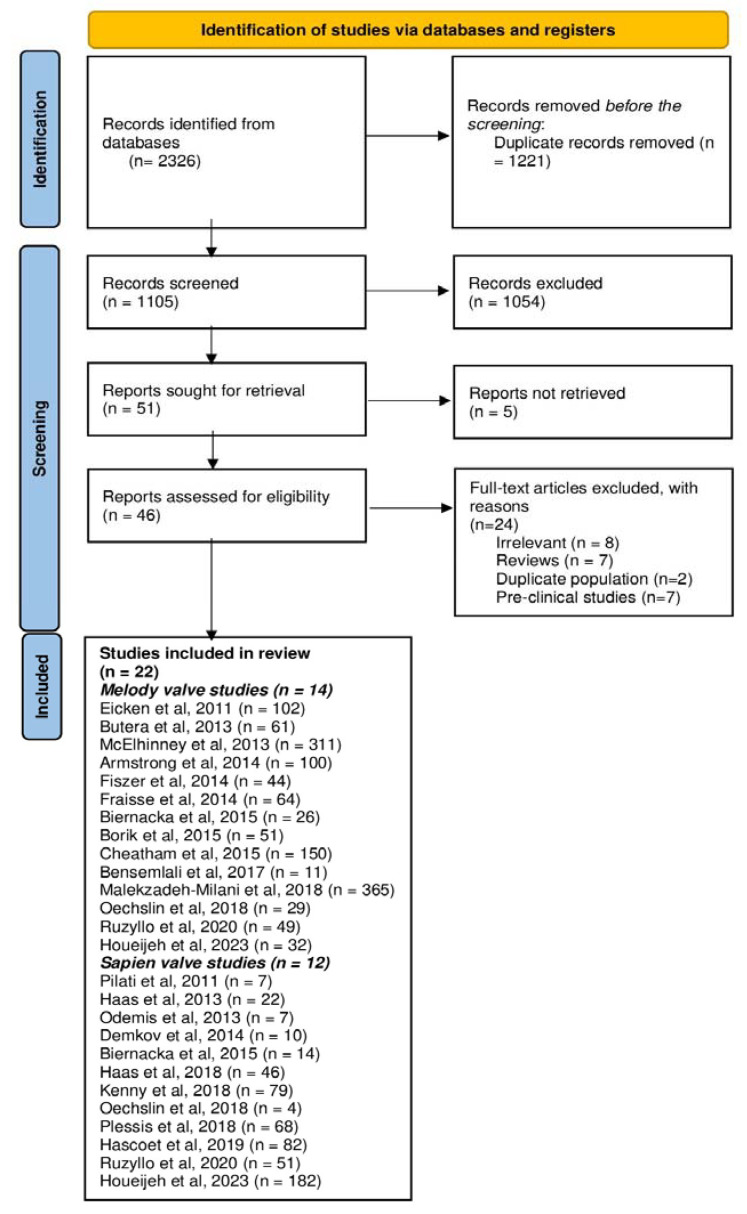
PRISMA flow showing the selection process of studies [11,13,21,22,23,24,25,26,27,28,29,30,31,32,33,34,35,36,37,38,39,40].

From a total of 2326 records that were identified in the literature search, 22 prospective observational studies (including 10 Melody valve studies, 8 Sapien valve studies, and 4 studies that included both valves (572 patients that used the Sapien valve and 1395 patients that used the Melody valve)) met the criteria for inclusion in the systematic review and meta-analysis [11,13,21,22,23,24,25,26,27,28,29,30,31,32,33,34,35,36,37,38,39,40] (Figure 2). Characteristics of all the included studies are presented in Table 1. 

**Figure 3:**
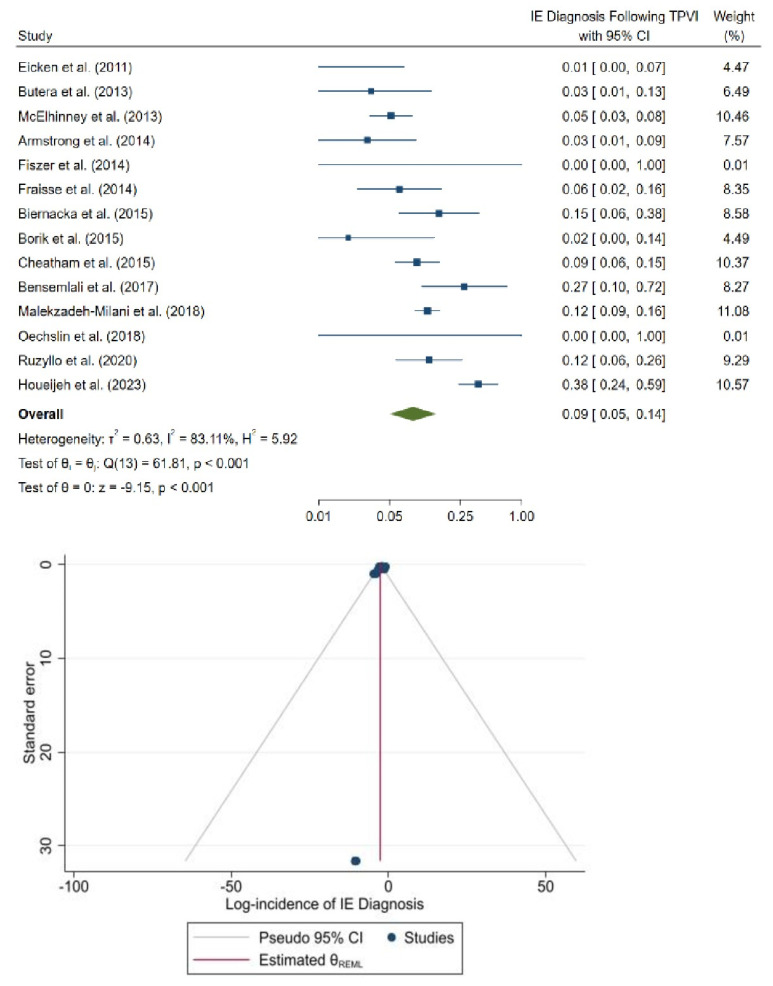
Forest plot (**top**) and funnel plot (**bottom**) of the incidence of endocarditis after Melody pulmonary valve implantation [11,26,28,30,31,32,33,34,35,36,37,38,39,40].

**Figure 4:**
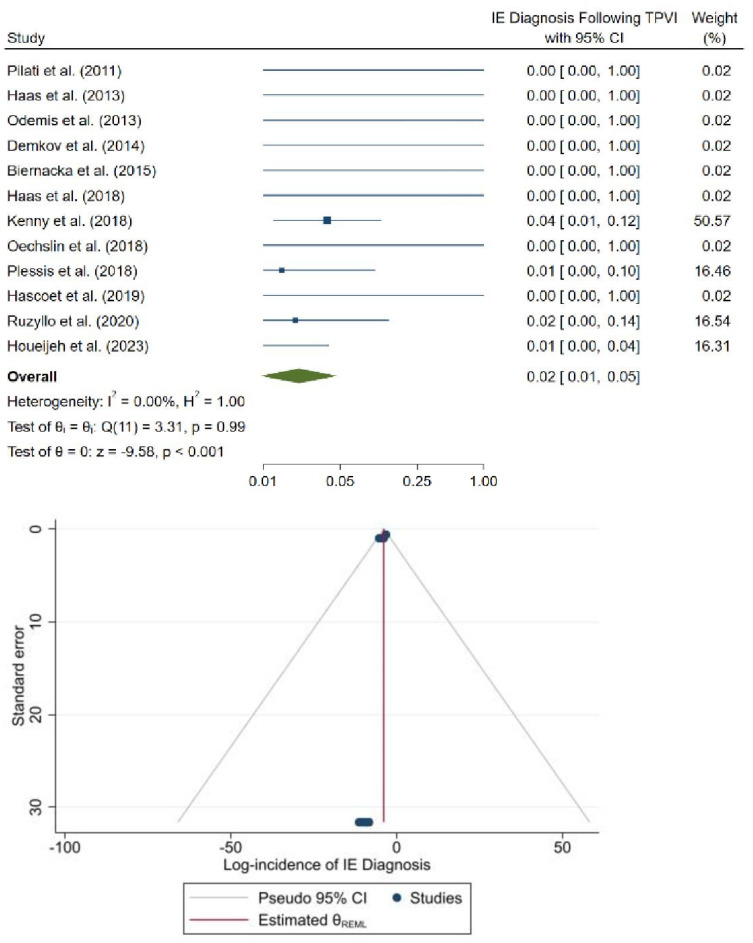
Forest plot (**top**) and funnel plot (**bottom**) of the incidence of endocarditis after Sapien pulmonary valve implantation [13,21,22,23,24,25,26,27,28,29,30,31].

The methodological quality of studies was assessed using the NOS for observational studies (Supplementary Table S1). Upon qualitative assessment, all the studies were of high quality, with NOS scores of 8–9. No indication of small-study bias was observed in the funnel plots (Figure 3 and Figure 4).

### 3.2. Incidence of IE Following TPVI

For the Melody valve, diagnosis of IE following TPVI was reported in 14 studies; 109 of 1395 patients received an IE diagnosis. In two studies (14.3%), zero patients were diagnosed with IE. The pooled incidence of IE diagnosis following TPVI was 8.5% with significant study heterogeneity (95% CI: 4.8% to 15.2%, *p* < 0.001, I^2^ = 83.1%; Figure 3).

For the Sapien valve, diagnosis of IE following TPVI was reported in 12 studies; 6 of 572 patients received an IE diagnosis. In eight studies (66.7%), zero patients were diagnosed with IE. The pooled incidence of IE diagnosis following TPVI was 2.1% with low study heterogeneity (95% CI: 0.9% to 5.1%, *p* < 0.99, I^2^ = 0.0%; Figure 4).

The meta-regression indicated that the Sapien valve had a 79.6% lower incidence of IE compared to the Melody valve (95% CI: 24.2% lower to 94.4% lower, *p* = 0.019), with valve type explaining 34% of study heterogeneity. Notably, length of follow-up was not significantly associated with the incidence of IE (RR: 1.02, 95% CI: 0.98 to 1.05, *p* = 0.306, R^2^ = 0.0%); as such, after controlling for length of follow-up, the Sapien valve retained a 78.8% lower adjusted incidence of IE compared to the Melody valve (95% CI: 21.7% lower to 94.2% lower, *p* = 0.022, R^2^ = 33.3%).

### 3.3. Sensitivity Analysis

Meta-analyses that include zero-event studies pose a statistical challenge, with traditional practice generally using a continuity correction or excluding zero-event studies [19,41,42]. However, this practice may bias estimates and impact practical conclusions [43,44]. Recent methodology suggests that Poisson, negative binomial, or binomial regression models may better estimate the aggregate effect in meta-analysis with zero-event studies [43,45,46,47,48]. As shown in Table 2, each method estimated similar pooled incidences across Sapien and Melody valves with inferences identical to those reported above for the primary analysis.

### 3.4. IE Treatment Characteristics

Following Melody valve implantation, the use of antibiotics was adequate for treatment in 45% of IE patients. Surgical explantation was necessary in 33% of cases, and transcatheter pulmonary valve intervention (redoing TPVI or transcatheter pulmonary valve explantation) was necessary in 14% of cases. About 8% of the patients with IE involving Melody valves died. There were just four instances where data are presented about the Sapien valves. In these IE patients who received Sapien valve implantation, the majority (75%) were treated with antibiotics, indicating a mild level of severity.

The microbial data showed Staphylococcus aureus was the most common pathogen involved. A total of 82 (78 Melody valve cases and 4 Sapien valve cases) occurrences mentioned the bacteria that caused them. Staphylococci were the cause of the majority (45%) of IE cases following Melody implantation. Among these, 20 (26%) involved S. aureus. A total of 24 (32%) of these cases were because of streptococci. Following Sapien valve implantation, staphylococci were the cause of majority (75%) of IE cases, of which 25% of the cases were due to Staphylococcus aureus. Patient and microbiological characteristics are presented in Table 3.

## 4. Discussion

The major finding of our systematic review and meta-analysis was that IE following TPVI was less prevalent with Sapien valves (pooled IE incidence of 2.1%) than after the implantation of a Melody valve (pooled IE incidence of 8.5%).

The pooled incidence of 8.5% with Melody valves noted in our study is comparable to that reported in the most recent studies. The higher incidence of IE seen with Melody valves compared to Sapien valves can be problematic to explain given that both valve systems use the same implantation methodologies and similar biological valve elements. However, there are some differences; the Sapien valve leaflets are made from bovine pericardium and are handled with a unique descaling and denaturing procedure that involves glutaraldehyde fixation to produce stable, durable, and biologically inert tissue that is suitable for implantation, in contrast to the Melody valve which is taken from a cow’s vein and then preserved without any processing [49]. The technique used for treating and preserving the Sapien valve may theoretically make it less likely for bacteria to invade it. Another possible explanation can be due to different blood flow patterns that have been seen to contribute to IE [40]. The Sapien valves’ larger diameters have led to speculation that there may be lower turbulence and, consequently, a reduced risk of thrombosis. Prior studies have shown an increasing gradient across the Melody valve, which can be caused by the thrombotic material inside the valves along with extensive granulocytic infiltrations [13,50,51].

Our findings are in accordance with prior studies by van Dijk et al. and Haas et al., who reported a higher risk of IE with bovine jugular valves (the Melody valve and Contegra graft) compared to other biological pulmonary valve substrates such as homografts and Sapien valves [13,15]. Similar findings were published by Mery et al., who showed that patients treated with Melody valves and Contegra grafts had a considerably higher incidence of late endocarditis than those treated with other valved conduits [52].

Furthermore, in an in vitro comparison of the bacterial adherence of Staphylococcus aureus and Streptococcus sanguinis strains to the biological substrates of the Sapien valve and the Melody valve, a substantial difference favoring the Sapien valve material was observed in a study by Jalal et al. [53]. They were able to show that bacterial adhesion was higher in control groups for S. aureus on the bovine jugular venous wall and also higher for S. sanguinis on the Melody valve leaflets. Additionally, adhesion was also significantly higher for both bacteria in traumatized Melody valve leaflets, suggesting that the Melody valve’s biological component is more susceptible to IE than other types of valve substrates. These in vitro results are consistent with those seen in our study, where we found a significant prevalence of blood cultures positive for staphylococci and streptococci among the included studies. Similar results were reported by Veloso et al. [54]. It is important to note that although many prior studies have shown sparse incidence of endocarditis with Sapien valves, the Sapien valve group had a shorter follow-up period and smaller patient numbers in many of the included studies [13,22,24,25,26,27,49,55,56,57]. Finally, our study’s findings are consistent with the most recent studies published by Rużyłło et al. [30] and Houeijeh et al. [31], who were able to show that patients that only received Melody valve implantations had a higher incidence of IE compared to those receiving Sapien valves.

Since it was first introduced, the Melody valve’s design has remained unchanged [58,59]. The Sapien valves have altered their external design according to the needs of the aortic valve market; however, the biological components were constant [58,59]. Initially, the Sapien valve was first released onto the market, followed by the Sapien XT with modified stent material and, ultimately, the Sapien X3 valve with a further modified stent design and exterior design. For pulmonary use, the Sapien and Sapien XT are currently approved. Sapien 3 is being utilized off-label for TPVI on a growing scale [58,59].

Multiple studies have demonstrated the post-interventional residual RVOT gradient to be a major determinant of long-term outcomes, including the risk of IE [9,60]. While data regarding the type of valves presenting risk factors for IE have been mixed, the majority of studies [13,16,17,26], including a large retrospective study published recently [61], have shown valve type as a possible risk factor for IE. Multiple published reviews and studies have shown an increasing number of IE events with Melody valves [13,16,17,26]. Additionally, most of the recent studies have clearly shown a trend favoring Sapien valves regarding the incidence of IE [13,16,17,26]. The residual RV-to-PA pressure gradient at the time of TPVI was one possible contributing factor to the elevated risk of IE for the Melody valve [11,62].

### Limitations

Our study has several limitations that warrant discussion. The data analyzed are from observational studies and single-arm studies, not randomized controlled trials. Only four multi-arm studies were identified during the literature search [26,28,30,31]. There is intrinsic heterogeneity between different studies in terms of the representation of baseline data, study design, and outcome measures. Although some of the studies included in this meta-analysis were matched according to baseline features, the other studies had significant differences in patient baseline characteristics between the two cohorts. There is also the possibility of publication bias among the outcomes where significant heterogeneity was observed [63,64,65,66]. We were unable to assess true publication bias as we do not have access to non-published studies. The major shortcoming of this study is that annualized IE incidence, as reported in prior studies, could not be calculated due to inaccessible patient-level data. While there is a significant difference in follow-up duration between the two valves, with longer follow-up for the Melody valve compared to the Sapien valve, this was addressed by controlling for follow-up in the meta-regression analysis, which showed that length of follow-up was not significantly associated with the incidence of IE. The dimensions of the RVOT determined which valve to use [30]. Melody valves, with a maximum available valve size of 22 mm, were implanted in patients with a narrow RVOT, whereas Sapien valves were chosen for patients with a large RVOT (landing zone of more than 22 mm) [30]. Smaller stenotic RVOTs result in higher RVOT gradients, especially when younger patients grow up, which can further affect the long-term outcomes [30]. Although the study reveals conclusive evidence that a rise in IE cases is linked to Melody valves, the claim is still dependent on diverse observational studies with occasionally very small sample sizes and zero-event studies. This limitation has been addressed in the sensitivity analysis. Therefore, even while the IE difference between the Sapien and Melody valves is statistically significant and represents clinically meaningful findings, the results of the meta-analysis are still insufficient to assess all safety-related topics. Multi-arm trials would be required to obtain a distinct picture of the efficiency and side effects of the two valves. Lastly, since only aggregate data (and not patient-level data) were used in this meta-analysis, it is subject to similar selection and confounding biases as the included studies themselves.

## 5. Conclusions

Considering the information provided by the present study, it appears that the Sapien valve may have a lower incidence of IE compared to the Melody valve when used for TPVI.

## Figures and Tables

**Table 1:** Study characteristics of the included investigations.

Study	Year	Region/ Country	Study Design	Enrollment Period	Number of Patients	Types of Valve Used	Events	Time to IE (Months)	Management and In-Hospital Outcome	Microorganism	Follow-Up Duration
Eicken et al. [32]	2011	Germany	Prospective, observational study, single-arm	2006 to 2010	102	Melody	1	6	SX = 1	Staphylococcus aureus = 1	12 months
Pilati et al. [23]	2011	Italy	Prospective, observational study, single-arm	2010 to 2011	7	Sapien	0	-	-	-	3 months
Butera et al. [33]	2013	Italy	Prospective, observational study, single-arm	2007 to 2010	61	Melody	2	5	SX = 2	Staphylococcus aureus = 2	30 months
Haas et al. [22]	2013	Germany	Prospective, observational study, single-arm	2007 to 2010	22	Sapien	0	-	-	-	6 months
McElhinney et al. [11]	2013	USA	Prospective, observational study, single-arm	2007 to 2009	311	Melody	16	16	ABX = 8, TI = 2, SX = 4, D = 2	Staphylococcus aureus = 5, other Staphylococcus = 5, Streptococcus = 6, other gram-negative bacteria = 1	24 months
Odemis et al. [24]	2013	Turkey	Prospective, observational study, single-arm	2007 to 2010	14	Sapien	0	-	-	-	30 months
Armstrong et al. [34]	2014	USA	Prospective, observational study, single-arm	2007 to 2010	100	Melody	3	3	ABX = 2, SX = 1	Staphylococcus aureus = 2, other gram-negative bacteria = 1	12 months
Demkow et al. [25]	2014	Poland	Prospective, observational study, single-arm	2011 to 2012	10	Sapien	0	-	-	-	2 months
Fiszer et al. [35]	2014	Poland	Prospective, observational study, single-arm	2009 to 2016	44	Melody	0	-	-	-	35 months
Fraisse et al. [36]	2014	France	Prospective, observational study, single-arm	2008 to 2010	64	Melody	4	26	SX = 3, D = 2	Other Staphylococcus = 2, Streptococcus = 1	54 months
Biernacka et al. [26]	2015	Poland	Prospective, observational study, multi-arm	2008 to 2012	26	Melody and Sapien	4	-	SX = 4, D = 1	-	20 months
Borik et al. [37]	2015	Canada	Prospective, observational study, single-arm	2005 to 2011	51	Melody	1	60	SX = 1	Other gram-positive bacteria = 1	54 months
Cheatham et al. [38]	2015	USA	Prospective, observational study, single-arm	2007 to 2014	150	Melody	14	-	ABX = 6, TI = 8, D = 1	-	54 months
Bensemlali et al. [39]	2017	France	Prospective, observational study, single-arm	2000 to 2015	11	Melody	3	-	ABX = 1, SX = 2	-	46 months
Haas et al. [13]	2018	Germany	Prospective, observational study, single-arm	2000 to 2015	46	Sapien	0	-	-	-	60 months
Kenny et al. [27]	2018	USA	Prospective, observational study, single-arm	2008 to 2014	79	Sapien	3	2	ABX = 3	Other Staphylococcus = 2, HACEK = 1	36 months
Malekzadeh-Milani et al. [40]	2018	France	Prospective, observational study, single-arm	2008 to 2016	365	Melody	43	31	ABX = 27, TI = 7, SX = 6, D = 3	Other Staphylococcus = 7, Staphylococcus aureus = 12, Streptococcus = 16, GP = 5, GN = 4	43 months
Oechslin et al. [28]	2018	Switzerland	Prospective, observational study, multi- arm	2008 to 2016	29	Melody and Sapien	0	-	-	-	43 months
Plessis et al. [29]	2018	France	Prospective, observational study, single-arm	2011 to 2017	71	Sapien	1	-	SX = 1	-	12 months
Hascoet et al. [21]	2019	France	Prospective, observational study, single-arm	2016 to 2018	82	Sapien	0	-	-	-	17 months
Rużyłło et al. [30]	2020	Poland	Prospective, observational study, multi-arm	2008 to 2019	49	Melody and Sapien	6	35	-	-	66 months
Houeijeh et al. [31]	2023	France	Prospective, observational study, multi-arm	2008 to 2020	32	Melody and Sapien	12	-	SX = 17	-	2.8 years

Note: Abbreviations—ABX: antibiotics only; D: Death; HACEK: Haemophilus species, Aggregatibacter species, Cardiobacterium hominis, Eikenella corrodens, and Kingella species; IE: infective endocarditis; SX: surgery; TI: transcatheter interventions (redo transcatheter pulmonary valve implantation or transcatheter pulmonary valve explantation).

**Table 2:** Sensitivity analysis: incidence of IE diagnosis stratified by method to address zero-event studies.

Method	Sapien	Melody	Incidence in Sapien vs. Melody	
	Incidence (95% CI) [%]	Incidence (95% CI) [%]	Risk Ratio (95% CI)	*p*
Primary Analysis	2.1 (0.9, 5.1)	8.5 (4.8, 15.2)	0.21 (0.06, 0.76)	0.019
Sensitivity Analysis				
0.5 Continuity Correction	2.6 (1.3, 5.2)	7.7 (4.3, 1.4)	0.32 (0.12, 0.82)	0.020
Exclusion of Zero-Event Studies	2.1 (0.6, 7.7)	8.5 (4.7, 15.4)	0.21 (0.05, 0.81)	0.026
Poisson Regression	0.4 (0.1, 2.2)	7.2 (2.8, 18.2)	0.06 (0.01, 0.30)	0.011
Binomial Regression	0.4 (0.1, 2.0)	7.3 (2.8, 18.8)	0.05 (0.01, 0.26)	0.010

Note: Melody is used as the reference valve. Hand calculation of relative risk reduction may vary from that presented due to rounding and the use of fixed vs. random-effects estimation. Meta-analysis of the Sapien valve used fixed-effects estimation, and meta-analysis of the Melody valve used random-effects estimation. Meta-regression used a random-effects model to account for between-study heterogeneity. Poisson regression with random intercept variance fit the data better compared to its fixed-effect variant (*p* < 0.001). Negative binomial regression was not necessary as it did not fit the data significantly better compared to Poisson regression. Binomial regression with random intercept variance better fit the data compared to binomial regression with fixed effects (*p* < 0.001).

**Table 3:** Patient characteristics and microbiological data.

	Melody	Sapien
Number of patients	1395	572
Mean age	20 ± 7	23 ± 6
Mean follow-up	33 ± 23 months	22 ± 18 months
IE events (cumulative incidence)	109 (7.8%)	6 (1%)
Time to IE	21 ± 18 months	2 ± 1 months
Management	*n* = 105	*n* = 4
Only antibiotic treatment	45%	75%
Explantation by surgery	33%	25%
Transcatheter pulmonary valve interventions (redoing TPV implantation or TPV explantation)	14%	–
In-hospital outcomes	*n* = 105	*n* = 4
Death	8%	–
Microbiological data	*n* = 78	*n* = 4
Staphylococcus aureus	26%	25%
Other staphylococci	19%	50%
Streptococcus	32%	–
Other gram-positive bacteria	10%	–
Other gram-negative bacteria	6%	–
HACEK	7%	25%

HACEK: Haemophilus, Aggregatibacter, Cardiobacterium, Eikenella, and Kingella; IE: infective endocarditis; TPV: transcatheter pulmonary valve.

## Data Availability

The data supporting this article are sourced from the public domain and are available in the articles cited throughout.

## Supplementary Materials

The following supporting information can be downloaded at: https://www.mdpi.com/article/10.3390/jcm12154886/s1, Table S1: The Newcastle-Ottawa scale assessing the quality of included studies.

## Abbreviations

CI	confidence intervals
CHD	congenital heart disease
IE	infective endocarditis
NOS	Newcastle–Ottawa Scale
PRISMA	Preferred Reporting Items for Systematic Reviews and Meta-Analyses
PPVI	percutaneous pulmonary valve implantation
RR	relative risk
RVOT	right ventricular outflow tract
TPVI	transcatheter pulmonary valve implantation
