# Supplementary material for: Infective Endocarditis Risk with Melody versus Sapien Valves Following Transcatheter Pulmonary Valve Implantation: A Systematic Review and Meta-Analysis of Prospective Cohort Studies
Source: J Clin Med. 2023 Jul 25;12(15):4886. doi: 10.3390/jcm12154886 (PMC10419461; doi:10.3390/jcm12154886)
Supplement: Supplementary file 1 [file jcm-12-04886-s001.zip › jcm-2383346-supplementary.pdf]

Supplementary Table S1. The Newcastle-Ottawa scale assessing the quality of included studies.

**Study**	**Selection**				**Comparability (Confounding)**	**Outcome**			**Total**
	Repres-ntative of exposed cohort	Selection of the non-exposed cohort	Ascertain-ment of exposure	Endpoint not present at start		Asse-ssment of Outcome	Follow-up duration	Adequ-acy follow-up	
**Eicken et al. [32]**	*	*	*	*	*	*		*	7
**Pilati et al. [23]**	*	*	*	*	*	*	*	*	8
**Butera et al. [33]**	*	*	*	*	**	*	*	*	9
**Haas et al. [22]**	*	*	*	*	*	*	*	*	8
**McElhinney et al. [11]**	*	*	*	*	*	*	*	*	8
**Odemis et al. [24]**	*	*	*	*	*	*	*	*	8
**Armstrong et al. [34]**	*	*	*	*	**	*	*	*	9
**Demkow et al. [25]**	*	*	*	*	*	*	*	*	8
**Fiszer et al. [35]**	*	*	*	*	**	*	*	*	9
**Fraisse et al. [36]**	*	*	*	*	*	*	*	*	8
**Biernacka et al. [26]**	*	*	*	*	**	*	*	*	9

**Borik et al. [37]**	*	*	*	*	**	*	*	*	9
**Cheatham et al. [38]**	*	*	*	*	*	*	*	*	8
**Bensemlali et al. [39]**	*	*	*	*	*	*	*	*	8
**Haas et al. [13]**	*	*	*	*	**	*	*	*	9
**Kenny et al. [27]**	*	*	*	*	**	*	*	*	9
**Malekzadeh-Milani et al. [40]**	*	*	*	*	*	*	*	*	8
**Oechslin et al. [28]**	*	*	*	*	**	*	*	*	9
**Plessis et al. [29]**	*	*	*	*	**	*	*	*	9
**Hascoet et al. [21]**	*	*	*	*	*	*	*	*	8
**Rużyłło et al. [30]**	*	*	*	*	**	*	*	*	9
**Houeijeh et al. [31]**	*	*	*	*	**	*	*	*	9

Notes: The Newcastle-Ottawa scale uses a star system (0 to 9) to evaluate included studies on 3 domains: selection, comparability, and outcomes. Star (*) = item presents. Maximum 1 star (*) for selection and outcome components and 2 stars (**) for comparability components. Higher scores represent higher study quality.
